# *Onosma fuyunensis* (Boraginaceae), a new species from Xinjiang, China

**DOI:** 10.3897/phytokeys.144.33287

**Published:** 2020-03-17

**Authors:** Yi He, Xue-Min Xu, Yu Zhou, Quan-Ru Liu

**Affiliations:** 1 Key Laboratory for Biodiversity Science and Ecological Engineering, Ministry of Education, College of Life Sciences, Beijing Normal University, Beijing 100875, China Beijing Normal University Beijing China; 2 Taiziwan School, Shenzhen, Guangdong 518067, China Taiziwan School Shenzhen China

**Keywords:** Boraginaceae, new species, *Onosma fuyunensis*, Xinjiang

## Abstract

*Onosma fuyunensis*, a new species of Boraginaceae from northern Xinjiang, China, is described and illustrated here. *Onosma fuyunensis* is similar to *O. simplicissima* and *O. gmelinii*; it differs in having a particularly bristly indumentum, unbranched stems, white and yellow corollas, anthers united only at base, and nutlets with a stipitate cicatrix. An updated key to the species of *Onosma* from Xinjiang and Altai Mountains is also provided.

## Introduction

*Onosma* L. (Boraginaceae-Lithospermeae), one of the largest genera in Boraginaceae, is primarily distributed in the temperate zones of the Old-World, with the main center of diversity in the Irano-Turanian region (Weigend et al. 2016). In recent years, several new species of *Onosma* have been described (Riedl et al. 2004, Binzet and Orcan 2007, Kandemir and Turkmen 2010, Aytac and Turkmen 2011, Almasi and Ranjbar 2015, Tarimcilar et al. 2015, Binzet 2016a, Binzet 2016b, Cecchi et al. 2016, Binzet and Eren 2018, Dehshiri 2018, He et al. 2018, Mehrabian and Mozaffarian 2018, Mehrabian and Rad 2018), which increases the total number of *Onosma* species to nearly 240. The northeastern region of the geographic distribution of the genus ranges from Turkestan to Altai (Johnston 1951), with the Altai Mountains running through Russia, China, Mongolia and Kazakhstan. In this area, four species and one subspecies of *Onosma* have been described (Krylov 1907, Popov 1953, Rybinskaya 1997, Baitulin and Kotukhov 2011, Urgamal et al. 2014). Furthermore, five species and one subspecies of *Onosma* are recorded in Xinjiang (Riedl 1995, Zhu et al. 1995, Pan and Nurbay 2004), a region of China that includes the southern part of Altai Mountains.

In Flora of the USSR, Popov (1953) provided the classification of sect. Aponosma DC. and sect. Euonosma DC (subsect. Haplotricha Boiss. and subsect. Asterotricha Boiss.), which was a combination of Candolle (1846) and Boissier (1875). On a sectional level, the morphology of the calyx in fruit and of the leaf indumentum are considered as the main diagnostic characters, but recent molecular data does not support the monophyly of these sections or subsections (Cecchi et al. 2016, Nasrollahi et al. 2019). On a specific level, the morphology of the flowers, the indumentum inside the corolla, and the morphology of the filaments and anthers have demonstrated to be useful characters (Johnston 1951, Liu 1989). Nutlet and pollen morphology also may be important characters to clarify similar species in *Onosma* taxonomy. (Binzet et al. 2014, Mehrabian et al. 2012, He et al. 2018).

In the process of a taxonomic revision of Chinese *Onosma* species, the identification of specimens from Xinjiang was extremely confusing, especially those specimens collected from Fuyun and Qinghe County, Altay City. Some of the specimens were identified as *O. simplicissima*, while others were assigned to *O. gmelinii*. However, within this group of *Onosma* is clearly another distinct taxon with a combination of characters that could not be associated with either *O. simplicissima* or *O. gmelinii*. Further detailed literature examination and field trips to Northern Xinjiang convinced us that this neglected taxon has been mistakenly mixed within those two species for more than half a century. To our best knowledge, it is not any other known species from the Altai Mountains and nearby regions. Here, we clarify the confusion by describing and illustrating this new species. An updated key of genus *Onosma* from Altai Mountains and Xinjiang is also provided for further study.

## Materials and methods

A total of 37 herbarium specimens of *Onosma fuyunensis* were collected from four populations in Northern Xinjiang, China in July, 2017. Type photos of accepted names and their synonyms from Xinjiang and adjacent regions were examined and compared along with 133 herbarium specimens from BNU, KUN, N, NAS, PE, XJA, XJBI, YUKU and 731 specimen pictures from BM, E, FL, K, KW, L, G, MO, MW, P and W. Images of morphological features were taken by Nikon digital camera with macro lens. Dried leaves, nutlets, and pollen grains were settled on stubs using double-sided adhesive tape and were coated with gold by Hitachi E-1045 ion sputter, photographed by Cam Scan Hitachi SU4800 Electron Microscope. For pollen studies, 30 pollen grains were measured for polar axis (P) and equatorial axis (E). Voucher information for the plant materials used was shown in Table 1. Terminology for pollen was used under Erdtman (1952) and Punt et al. (1994). The main characters for comparison of related species are presented in Table 2, which were measured by Image J 1.52a (Abràmoff et al. 2004). Conservation assessments were made according to the IUCN Standards and Petitions Committee (2019) guidelines.

**Table 1.** Voucher information for the plant materials used.

Taxa	Voucher information	Locality
*O. fuyunensis*	Y. He & Y. Zhou XJ133 (BNU)	China, Xijiang, Fuyun
*O. gmelinii* (corolla and pollen)	Anonymous 80617-1 (XJU)	China, Xijiang, Hebukesaier
*O. gmelinii* (nutlet)	Anonymous 803220 (XJU)	China, Xinjiang, Buerjin
*O. simplicissima*	Anonymous 19492 (YUKU)	USSR, Voronezh

**Table 2.** Comparison of *Onosma fuyunensis* with *O. gmelinii* and *O. simplicissima*.

Organ	Character	*O. fuyunensis*	*O. gmelinii*	*O. simplicissima*
Habit	life form	perennial herb with rosettes	perennial herb with rosettes	subshrub with woody branching base and sterile shoots, without rosettes
Leave	indumentum	spreading bristles	spreading bristles	appressed bristles
	venation	reticulate	obscure	obscure
Inflorescence	length (cm)	slightly elongating and straightening in fruit, 5–11	markedly elongating and straightening in fruit, 10–22	markedly elongating and straightening in fruit, 5–9
Bract	shape	lanceolate to linear-oblanceolate	lanceolate	lanceolate to linear-oblanceolate
	size (mm)	12–20 × 1.2–4.5	13–31 × 3.5–10	7–15 × 1.5–3.5
Calyx	lobe	parallel in fruit	converging in fruit	angular in fruit
	size (mm)	15–23 × 1–2	13–22.5 × 1.5–3	6–13 × 0.8–1.2
Corolla	length (mm)	22–27	19–30	15–20
	color	cream and light yellow	pale yellow	cream and light yellow
Androecium	anther (mm)	united only at base, included, 7–8	united into a tube, apex exserted, 8–10	united only at base, included, ca. 5
	filament (mm)	9–11	7–9	ca. 8
	pollen shape	isopolar	heteropolar	unknown
	polar axis (μm)	21.91 ± 1.19	14.41 ± 1.03	unknown
	equatorial axis (μm)	13.83 ± 0.17	9.98 ± 0.12	unknown
Nutlet	length (mm)	ca. 5	ca. 5	ca. 2.5
	cicatrix	stipitate	complanate	unknown
	epidermis cells	rectangular	reticulate	unknown

## Taxonomic treatment

### Onosma fuyunensis

Taxon classificationPlantaeBoraginalesBoraginaceae

Y. He & Q.R. Liu sp. nov.

0497B5A6-F75F-5A93-9221-8F830CDD36B0

urn:lsid:ipni.org:names:77208267-1

**Figure 1.**
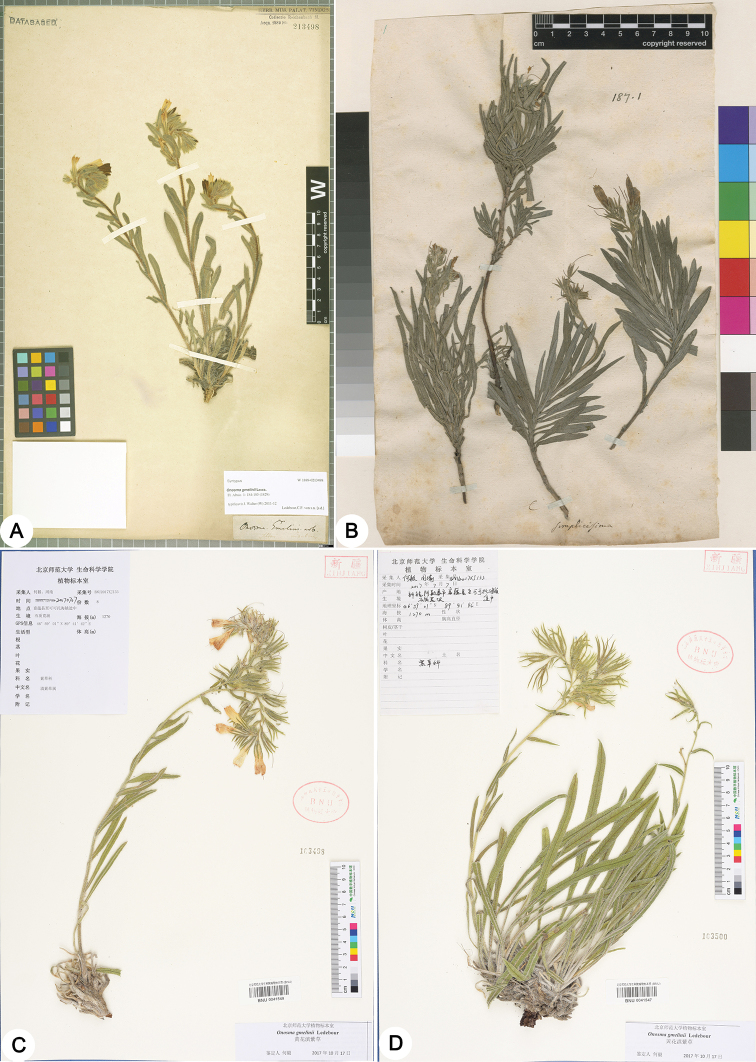
Type specimens of *Onosma gmelinii* (**A** syntype, W 1899-0213498), *O. simplicissima* (**B** lectotype, LINN No. 187.1) and *O. fuyunensis* (**C** holotype, BNU 0041549 **D** isotype, BNU 0041547).

**Figure 2.**
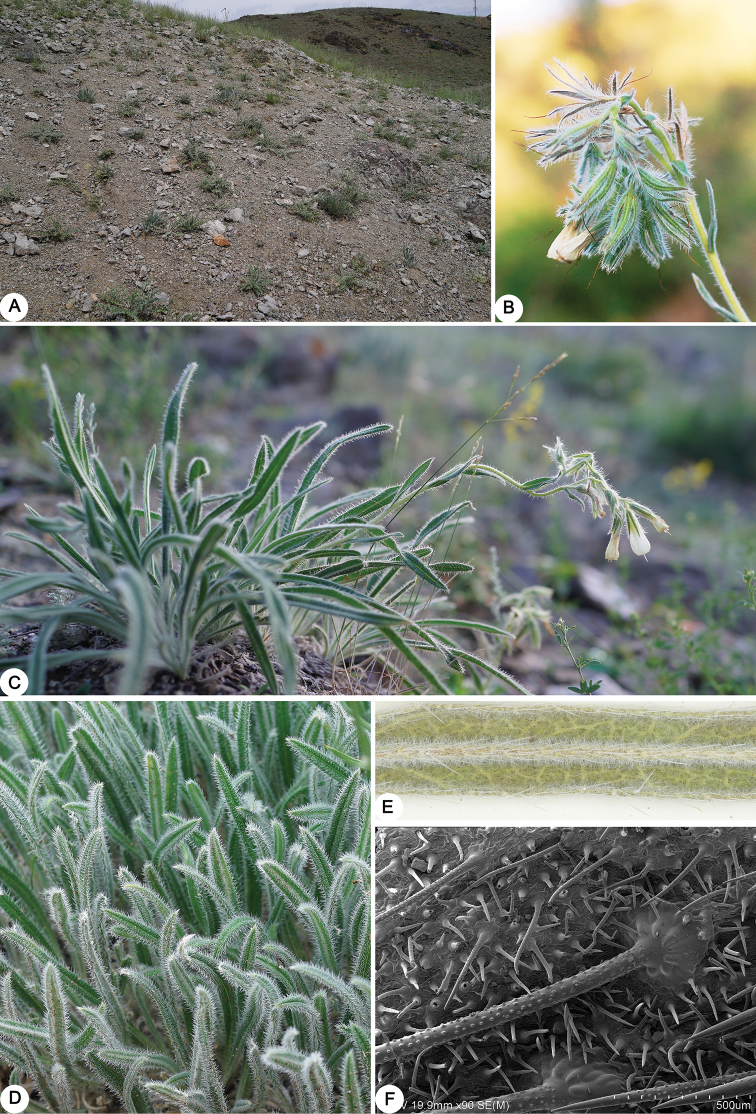
Photographs of *O. fuyunensis*. **A** Habitat **B** inflorescence during late flowering season **C** habit **D** basal leaves (show spreading bristles) **E** leaves in abaxial view (show netted venation) **F** scanning electron micrographs of leaves in adaxial view. Photo by Yi He.

**Figure 3.**
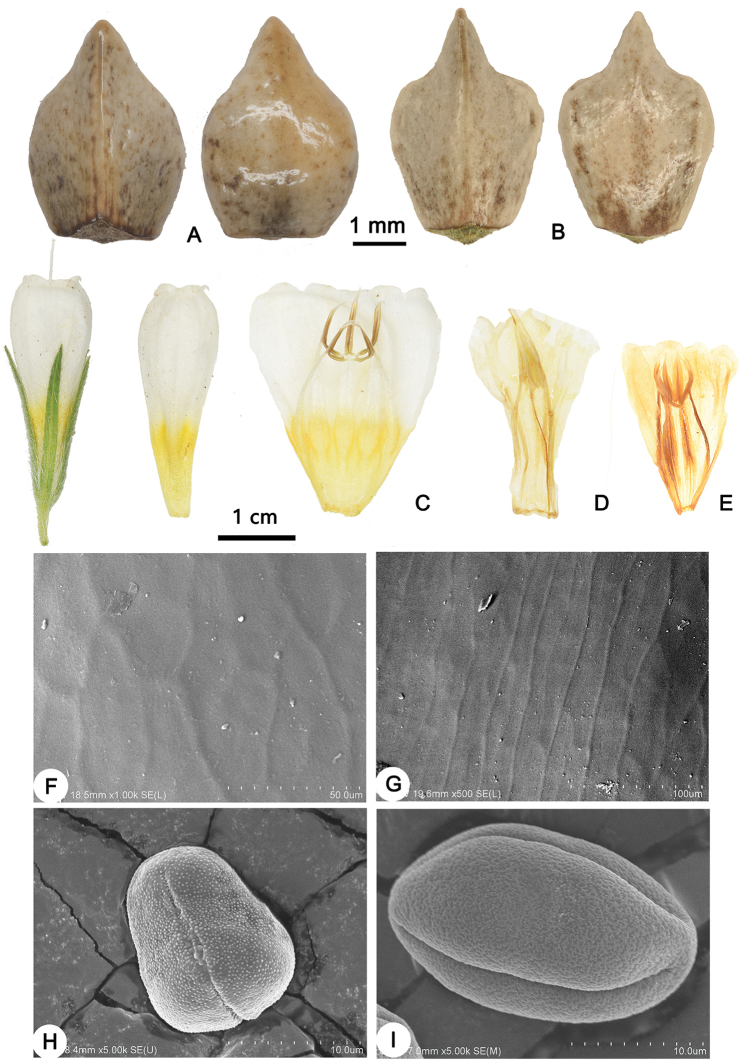
Characters comparison of *O. fuyunensis* and related species **A** nutlets of *O. gmelinii* (in adaxial and abaxial view) **B** nutlets of *O. fuyunensis* (in adaxial and abaxial view) **C** flowers of *O. fuyunensis***D** corolla of *O. gmelinii***E** corolla of *O. simplicissima***F** scanning electron micrograph of nutlets of *O. gmelinii***G** scanning electron micrograph of nutlets of *O. fuyunensis***H** scanning electron micrograph of pollens of *O. gmelinii***I** scanning electron micrograph of pollen of *O. fuyunensis*. Photo by Yi He.

**Figure 4.**
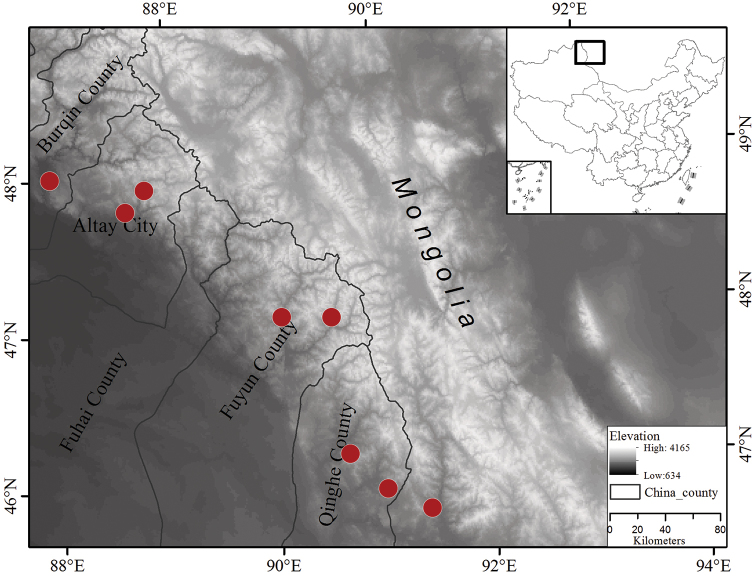
Distribution map of *O. fuyunensis*. Red points denote localities. Illustrated by Feng Xue and Yi He.

Figs 1C, D , 2 , 3B, C, G, I , 4

Onosma gmelinii auct. non Ledeb.: Fl. Reipub. Popul. Sinicae 64(2): 54. 1989. p.p.; Fl. China 16: 352. 1995. p.p.; Clavs Plantarum Xijiangensis. 428. 2000. p.p.; Fl. Xinjiangensis 4: 157. pl. 50. 2004. p.p.
Onosma simplicissima auct. non L.: Fl. China 16: 351. 1995; Fl. Xinjiangensis. 4: 157. 2004.

#### Type.

China. Xinjiang: Between Fuyun County and Keketuohai Town, *Y. He & Y. Zhou BNU2017XJ133*, 7 July 2017, 1270 m a.s.l., rocky slopes, 46°59'01"N, 89°41'42"E. (Holotype: BNU 0041549; Isotype: BNU, PE).

#### Diagnosis.

Closely allied to *O. simplicissima* L., a widespread species distributed from E Europe to E Siberia. It is differentiated by being perennial herb with rosettes (v.s. mostly subshrub with woody branching base and sterile shoots, Fig. 1B), having leaves with spreading bristles (Fig. 2 C–F, v.s. densely silky appressed pilose), larger nutlets (ca. 5 mm v.s. 2.5–3 mm), longer calyx (15–22 mm v.s. 6–13 mm) and corolla (22–27mm v.s. 18–20 mm). Also nearly to *O. gmelinii* Ledeb., but different through having obvious reticulate venation (v.s. obscure lateral veins), slightly elongating and straightening inflorescences in fruit (v.s. markedly elongating and straightening), longer and parallel calyx lobes in fruit (1.2–2 mm v.s. ca. 4mm, lobes converging), cream and pale yellow corolla (v.s. pale yellow), included anthers united only at base (v.s. united into a tube, Fig. 3D), nutlet with stipitate cicatrix and elongated, rectangular surfaces epidermis cells (v.s. complanate cicatrix and reticulate cells, Fig. 3A, F) and isopolar pollen grains (v.s. heteropolar, Fig. 3H).

#### Description.

Herbs perennial, 15–40 cm tall, hispid, strigose. Stems single or several (1–4) arise from rosettes, caspitose, erect, not branched, usually pale straw to light brown, densely covered with long white spreading bristles. Basal leaves short petiolate, linear to linear-oblanceolate, 10–23 cm × 3–10 mm, abaxially densely pubescent and hispid along rised midrib and margin, reticulate venation, adaxially densely appressed hispid and short strigose, base attenuate, apex acute; Cauline leaves sessile, lanceolate, 2–5 cm × 1.5–5 mm. Inflorescences terminal, solitary or dichotomously branched, 4–8 cm wide at anthesis, length to 11 cm in fruit, flowers 5–20; bracts lanceolate to linear-oblanceolate, 1.2–2 cm × 1.2–4.5 mm, densely hispid, short strigose. Pedicel short, ca. 5 mm. Calyx 1.5–2.3 cm × 1–2 mm, densely hispid, short strigose, parted nearly to base; lobes linear. Corolla cream above middle, light yellow below middle, clavate, 2.2–2.7 cm, base ca. 2 mm wide, gradually expanded upward; throat ca. 5 mm wide, obscurely pubescent outside, glabrous inside; lobes broadly triangular, ca. 1.5 × 3 mm. Filaments subulate, 9–11 mm, decurrent; anthers united only at base, 7–8 mm, included, apex sterile, ca. 2 mm. Style 2.4–2.8 cm, glabrous. Nectary ca. 1 mm, glabrous. Pollen grains isopolar, tricolporate and prolate, polar axis (P) 21.91 ± 1.19 μm, equatorial axis (E) 13.83 ± 0.17 μm, P/E ratio 1.58. Nutlets gray-brown, ca. 5 mm × 3 mm, lustrous, smooth, ventral keeled, stipitate cicatrix.

#### Phenology.

Flowering and fruiting occurs from May to July.

#### Etymology.

The specific epithet of the new species refers to its type locality, Fuyun County, Xinjiang, China.

#### Distribution and habitat.

*Onosma fuyunensis* is mainly distributed in Fuyun County, Qinghe County and Altay Prefecture (Fig. 4), it is also known from W Mongolia near the border (Khovd aimag), according to the photo record by Peter Kosachev (http://www.plantarium.ru/page/image/id/128255.html). It prefers dry rocky screes and upland meadows along the hillside, from 500–1400 m a.s.l. Species growing nearby are: *Echinops gmelinii* Turcz, *Goniolimon speciosum* (L.) Boiss., Artemisia rutifolia var. altaica (Kryl.) Krasch. and *Carex turkestanica* Regel.

#### Conservation status.

According to current data, *Onosma fuyunensis* grows in a large area of ca. 70, 000 km^2^ between N Xinjiang and W Mongolia. Similar habitats are common in this area. During our field investigation, four large populations (at least 50 mature individuals) of this species were easily found even at the end of its flowering season. *Onosma fuyunensis* could be the dominant species in some screes and meadows. In this area, human activities are infrequent, and grazing pressure is low. Historical specimens of this taxon are also abundant (from 16 different locations). According to the IUCN Standards and Petitions Committee (2019) criteria, we justify a preliminary status of ‘Least Concern’ (LC). More accurate quantitative analyses should be used for assessment after more field works in the future.

#### Additional specimens examined.

**China. Xinjiang**: Altay Prefecture, Dahe forestry station, 1400 m, 5 July 1985, *Anonymous 85-5751* (XJU00016072B); Altay Prefecture, Dahe forestry station, 1200 m, 5 July 1985, *Anonymous 85-0225* (XJU00016073B); Altay Prefecture, Dahe forestry station, 1400 m, 12 June 1985, *Pi 85018* (XJU00016076B); Altay Prefecture, Dahe forestry station, 900 m, 6 July 1985, *Pi 85017* (XJU00016086B); Altay Prefecture, Aweitan Police checkpoint, 815 m, 8 July 2017, *Y. He et Y. Zhou BNU2017XJ153* (BNU0041544); Altay Prefecture, Xiaodonggou forest park, 1000 m, 8 July 2017, *Y. He et Y. Zhou BNU2017XJ159* (BNU0041543); Altay Prefecture, Xiaoxigou, 1 July 1973, *Anonymous Altay197* (XJBI00031718); Fuyun County, roadside to Qinghe County, 1200 m, *G.J. Liu et al. Altay901* (XJBI00031717); Altay Prefecture, suburban areas, 900–950 m, 29 May 1987, *X.Y. Chen et Q.X. Liu 87061* (NAS00214133); Altay Prefecture, Television tower mountain, 780 m, 30 May 1991, *Z.J. Ma* # (XJA00058804); Altay Prefecture, Dahe forestry station, 900 m, 10 June 1984, *C.Y. Yang 84008* (XJA00058805); Altay Prefecture, suburban areas, 800 m, 6 June 1989, *B. Wang 89-0038* (XJA00058808); Fuyun County, Akequtadao Mountain, 1200 m, 8 June 1959, *Xinjiang Expedition Team 10417* (XJBI00031710, XJBI00031711, PE01354749); Fuyun County, 73 km roadside to Fuhai County, 850 m, 5 June 1974, *Anonymous 00959* (XJBI00031714; XJBI00031715); Fuyun County, 1100 m, 9 July 1977, *Anonymous 11247* (XJBI00031713); Fuyun County, Kemuqi, 800 m, 22 August 1965, *Anonymous 652179* (XJBI00031712); Fuyun County, hydropower station, 750 m, 9 July 1988, *X.Y. Chen et Q.X. Liu 88210* (N138252360, N138252361); Qinghe County, road side from Areletuobie to town, 1170 m, 10 July 2017, *Y. He et Y. Zhou BNU2017XJ083* (BNU0041564); Qinghe County, Kuosirele Village, Areletuobie town, 1143m, 6 July 2017, *Y. He & Y. Zhou BNU2017XJ089* (BNU0041561); Qinghe County, near the checkpoint, 1140m, 7 July 2017, *Y. He et Y. Zhou BNU2017XJ123* (BNU0041554); Qinghe County, Buergen Beaver National Nature Reserve, 11 June 1989, *B. Wang 89-153* (XJA00058826); Buerjin County, Gaochao Commune, 500 m, August 1972, *C.Y. Yang A720295* (XJA00058802, XJA00058803).

## Discussion

This species is widely distributed in the middle and low altitude mountains in the eastern part of the Altai Mountains. In the past 60 years, multiple specimens of this taxon have been collected; however, they were not recognized correctly.

According to its nature of indumentum, *O. fuyunensis* belongs to subsect. Haplotricha Boiss. Morphologically, in the color and shape of the corolla, this species is close to *O. simplicissima*. The upper part of the fresh corolla is milky white, and the part below the calyx is light yellow. The whole corolla turns pale yellow after drying. The filaments are slightly longer than the anthers, which are united only at base and not exserted from the corolla. The calyx of this species is longer, nearly half to 2/3 of the length of the corolla, while the calyx of *O. simplicissima* is shorter, only ca. 1/3 of the length of the corolla. There is a large difference in the vegetative features of the plants of this newly described species. *O. fuyunensis* is perennial herb with highly-developed rosettes, lacking sterile shoots, covered with long and spreading bristles, while *O. simplicissima* is subshrub with differentiation of flowering shoots and sterile shoots (without rosettes) and its indumentum is appressed. The vegetative parts of *O. fuyunensis* are similar to those of *O. gmelinii*. Both of these species are perennial herbs with rosettes and spreading bristles. The species could not be easily distinguished without the presence of cymes. In addition to the significant differences in aforementioned floral morphology, the stems of *O. gmelinii* are usually bluish, and those of *O. fuyunensis* are generally straw-colored to light-brown.

Geographically, *O. fuyunensis* is mainly distributed in the southeastern part of the Altai Mountains in China and Mongolia. *O. simplicissima* was recorded in Northern Xinjiang by Zhu et al. (1995) and Pan and Nurbay (2004); however, after we examined multiple specimens *of Onosma* collected from China, no specimen should be identified to this taxa. Those previous records were misidentified as either *O. fuyunensis* or *O. gmelinii*. According to Popov (1953), *O. simplicissima* can range eastward to the upper reaches of the Yenisei River and to the northern part of Kazakhstan, so there is no overlap between these two species. *O. gmelinii* is primarily distributed in Central Asia and Siberia. In China, this species is distributed from Kanas Lake, the junction of China and Kazakhstan, to Qinghe County, which makes it sympatric with *O. fuyunensis*.

### Key to the species of genus *Onosma* in Altai Mountains and Xinjiang

**Table:** 

1	Anthers coherent only at base	**2**
–	Anthers coherent into a tube	**6**
2(1)	Subshrubs or perennial herbs, stems mostly not branched; corolla cream and pale yellow, filaments longer than anthers	**3**
–	Biennial herbs; stems branched; corolla yellow, filaments shorter than anthers	**4**
3(2)	Perennial herbs with rosettes, leaves with spreading bristles, calyx lobes parallel in fruit, 15–23 mm, corolla 22–27 mm	**1. *O. fuyunensis***
–	Mostly subshrubs with sterile shoots, leaves with appressed bristles, calyx lobes angular in fruit,6–13 mm corolla 15–20 mm	**2. *O. simplicissima***
4(2)	Plants strongly whitish gray hirsute; corolla longer than 20 mm	**3. *O. setosa***
–	Plants yellow-green hirsute or sparse whitish hirsute; corolla shorter than 20 mm	**5**
5(4)	Cauline leaves lanceolate, 4–6 cm × 6–11 mm	**4. *O. borysthenica***
–	Cauline leaves linear, 3–5 cm × 3–5 mm	**5. O. setosa subsp. transrhymnensis**
6(1)	Bracts longer than calyx	**6. *O. apiculata***
–	Bracts not longer than calyx	**7**
7(6)	Plants covered with long horizontally spreading bristles; corolla slightly longer than calyx	**7. *O. irritans***
–	Plants covered with shorter bristles; corolla twice as long as calyx	**8. *O. gmelinii***

## Supplementary Material

XML Treatment for Onosma fuyunensis
